# Caractéristiques cliniques et évolution hospitalière des patients sous traitement antithrombotique dans une unité de soins intensifs cardiologiques d'Afrique subsaharienne: étude rétrospective comparative à l'institut de cardiologie d'Abidjan

**DOI:** 10.11604/pamj.2026.54.37.52294

**Published:** 2026-06-09

**Authors:** Christelle Gbassi, Florent Koffi, Cédric Kee, Esther Ehouman, Stéphane Kouame, Adon Djoma, Hermann Yao, Esaie Soya, Arnaud Ekou, Ami Avoh, Djinguin Koffi, Djeneba Bamba-Kamagaté, Christophe Konin

**Affiliations:** 1 Institut de Cardiologie d'Abidjan, Abidjan, Côte d'Ivoire,; 2 Centre Hospitalier et Universitaire, Bouaké, Côte d'Ivoire

**Keywords:** Antithrombotiques, hémorragie, soins intensifs, Afrique subsaharienne, Antithrombotic therapy, hemorrhage, intensive care, sub-Saharan Africa

## Abstract

**Introduction:**

les traitements antithrombotiques sont indispensables dans la prise en charge des affections cardiovasculaires, mais exposent à des complications hémorragiques. Cette étude visait à décrire l'utilisation des antithrombotiques et les complications associées en unité de soins intensifs cardiologiques, et à analyser l'évolution de leur prescription dans le temps.

**Méthodes:**

étude observationnelle rétrospective, descriptive, analytique, incluant 1000 patients hospitalisés en unité de soins intensifs à l'Institut de Cardiologie d'Abidjan, ayant reçu au moins un traitement antithrombotique, sur une période de 12 ans. Les données ont été collectées en deux phases, de 2013 à 2017, puis de 2018 à 2024, permettant l'analyse des tendances temporelles.

**Résultats:**

l'âge moyen était de 59,5±14,1 ans. Le facteur de risque cardiovasculaire prédominant était l'hypertension artérielle, le syndrome coronarien aigu constituait la principale indication du traitement antithrombotique. La proportion de patients sous antithrombotique a significativement augmenté entre les deux phases de l'étude (76,5% vs 78,7%, p=0,004). L'utilisation des fibrinolytiques a augmenté significativement entre les deux phases de l'étude (2,6% vs 6,6%). Les accidents aux antithrombotiques étaient surtout hémorragiques avec des facteurs indépendants incluant l'âge élevé (>65 ans), les fibrinolytiques et l'utilisation concomitante de plusieurs antithrombotiques. La mortalité hospitalière était plus élevée chez les patients avec un accident hémorragique, sans différence significative entre les deux périodes.

**Conclusion:**

les antithrombotiques restent fréquemment utilisés en unité de soins intensifs cardiologiques en Afrique subsaharienne, avec une tendance à l'augmentation des prescriptions. Une surveillance clinique et paraclinique rigoureuse est essentielle pour améliorer le pronostic vital des patients.

## Abstract (en)

**Introduction:**

antithrombotic therapies are essential in the management of cardiovascular diseases but carry a risk of bleeding complications. This study aimed to describe the use of antithrombotic agents and associated complications in a Cardiac Intensive Care Unit, and to analyse trends in their prescription over time.

**Methods:**

we conducted a retrospective, observational, descriptive, and analytical study including 1,000 patients admitted to the Intensive Care Unit of the Institute of Cardiology of Abidjan who received at least one antithrombotic therapy over 12 years. Data were collected in two phases, from 2013 to 2017 and from 2018 to 2024, allowing for the analysis of temporal trends.

**Results:**

the mean age was 59.5 ± 14.1 years. Hypertension was the most prevalent cardiovascular risk factor, and acute coronary syndrome was the main indication for antithrombotic therapy. The proportion of patients receiving antithrombotic treatment increased significantly between the two study periods (76.5% vs 78.7%, p = 0.004). The use of fibrinolytic agents also increased significantly (2.6% vs 6.6%). Adverse events related to antithrombotic therapy were predominantly hemorrhagic, with independent risk factors including advanced age (>65 years), use of fibrinolytics, and concomitant use of multiple antithrombotic agents. In-hospital mortality was higher in patients with hemorrhagic events, with no significant difference between the two periods.

**Conclusion:**

antithrombotic therapies remain widely used in Cardiac Intensive Care Units in sub-Saharan Africa, with an increasing trend in prescriptions. Rigorous clinical and laboratory monitoring is essential to improve patient outcomes.

## Introduction

Les maladies cardiovasculaires constituent la première cause de morbidité et de mortalité dans le monde [1]. En Afrique subsaharienne, leur prévalence connait depuis plusieurs décennies une progression rapide, en lien avec la transition épidémiologique. Elles figurent désormais parmi les principales causes de morbi-mortalité sur le continent et représentent désormais un enjeu majeur de santé publique [2]. Cette évolution s'accompagne d'une modification des pratiques thérapeutiques, avec un recours croissant aux traitements antithrombotiques [3]. Ces derniers sont indiqués tant à titre préventif que curatif dans de nombreuses situations cliniques, notamment au cours des syndromes coronariens aigus, de la maladie veineuse thromboembolique, des accidents vasculaires cérébraux ischémiques, des thromboses intracardiaques et de la fibrillation atriale [4,5]. Ces traitements ont connu une avancée majeure, avec l'apparition ces dernières années de nouvelles molécules ayant permis d'améliorer le pronostic des patients: les anticoagulants oraux directs [6,7].

Cependant, l'utilisation des antithrombotiques n'est pas sans risque, les complications hémorragiques demeurant une problématique majeure pouvant engager le pronostic vital des patients. En Afrique subsaharienne, les données relatives à l'usage des antithrombotiques en milieu hospitalier et surtout en unité de soins intensifs cardiologiques restent limitées, alors que les spécificités du contexte (conditions de surveillance clinique et biologique, profil des patients) peuvent influencer les pratiques cliniques. En Côte d'ivoire, peu de données hospitalières sur la gestion des médicaments antithrombotiques sont disponibles.

Cette étude visait donc à combler cette lacune en décrivant les caractéristiques cliniques des patients ayant reçu un traitement antithrombotique en cours d'hospitalisation en Unité de Soins Intensifs Cardiologiques (USIC) dans un pays d'Afrique subsaharienne, en identifiant les types et modalités d'utilisation de ces traitements, en évaluant l'évolution temporelle des prescriptions et des complications liées à leur utilisation et enfin, en analysant les facteurs associés à la survenue de ces complications.

## Méthodes

**Type d'étude:** il s'agissait d'une étude observationnelle monocentrique, rétrospective à visée descriptive et analytique.

**Cadre d'étude:** l'étude s'est déroulée de janvier 2013 à décembre 2024 dans l'unité de soins intensifs cardiologiques (USIC) de l'Institut de Cardiologie d'Abidjan (ICA), centre tertiaire de référence pour la prise en charge des affections cardiovasculaires en Côte d'Ivoire. La collecte des données a concerné 12 années réparties en deux phases successives: une première phase (phase 1) allant de janvier 2013 à décembre 2017 et une seconde phase (phase 2) de janvier 2018 à décembre 2024. Cette organisation a permis d'analyser l'évolution des pratiques de prescriptions ainsi que des complications liées aux traitements antithrombotiques au cours du temps.

**Population d'étude:** ont été inclus les patients hospitalisés en unité de soins intensifs de l'Institut de Cardiologie d'Abidjan pour une affection cardiovasculaire durant la période d'étude et ayant reçu au moins un traitement antithrombotique durant cette hospitalisation. La sélection des patients participants a été réalisée rétrospectivement à partir des dossiers des patients hospitalisés durant la période d'étude. Les dossiers incomplets avec une absence de données sur le traitement reçu et les dossiers de patients hospitalisés pour des pathologies non cardiovasculaires n'ont pas été inclus.

**Variables étudiées:** les variables étudiées ont été définies en lien avec les objectifs de l'étude. Le traitement antithrombotique a ainsi été défini, comme toute médication prescrite pour prévenir ou traiter la formation de caillots sanguins incluant les anti-agrégants plaquettaires (aspirine, clopidogrel, ticagrelor), les anticoagulants injectables (héparines, fondaparinux) ou oraux (antivitamines K, anticoagulants oraux directs) et les fibrinolytiques (altéplase, streptokinase). Pour ces traitements, les variables étudiées comprenaient le type de traitement reçu par le patient, le nombre de traitements antithrombotiques et la durée de traitement. A été considérée comme complication hémorragique (variable dépendante) dans le cadre de cette étude, tout saignement survenant chez un patient sous traitement antithrombotique, qu'il soit majeur ou mineur. L'hémorragie majeure a été définie selon les critères de l'International Society on Thrombosis and Haemostasis (ISTH) comme une hémorragie entraînant le décès, une hémorragie symptomatique critique (cérébro-méningée, oculaire), une hémorragie entraînant une transfusion ≥2 unités de sang ou une perte sanguine significative, une hémorragie nécessitant une intervention chirurgicale ou endovasculaire urgente. Les hémorragies mineures étaient définies par tout saignement clinique ne répondant pas aux critères d'une hémorragie majeure (par exemple: épistaxis, gingivorragie, hématome cutané). Les variables explicatives et facteurs de confusion potentiels tels que l'âge, le sexe, les facteurs de risque cardiovasculaire, le type et le nombre de traitements antithrombotiques ont été retenus pour l'analyse.

**Sources de données et méthodes de mesure:** les données ont été collectées de façon rétrospective, à partir de dossiers médicaux papier et électroniques des patients hospitalisés durant la période d'étude. Les informations ont été extraites à l'aide d'une fiche de collecte de données standardisée incluant les données sociodémographiques (âge, sexe, profession, couverture sociale), cliniques (facteurs de risque cardiovasculaire et thromboembolique, diagnostics), thérapeutiques (types de traitement, nombre d'antithrombotiques, durée du traitement) et évolutives (complications, mortalité hospitalière). Afin d'assurer la comparabilité entre les deux périodes, les mêmes définitions des variables d'intérêt ont été considérées et la même fiche de collecte des données a été utilisée, permettant ainsi d'assurer la cohérence des analyses.

**Biais:** cette étude présente certains biais inhérents à son caractère rétrospectif et monocentrique, notamment un biais de sélection et un biais d'information. Ceci peut limiter la généralisation des résultats à d'autres populations. Le biais d'information a cependant été minimisé grâce à l'utilisation d'une fiche de collecte standardisée et à des définitions de variables uniformes sur toute la collecte de données. De plus, l'échantillonnage stratifié par année a été utilisé pour limiter le biais de sélection et permettre une bonne représentativité temporelle.

**Taille de l'échantillon:** la taille minimale de l'échantillon pour la première période (2013-2017) avait été calculée dans le cadre d'une thèse précédente [8] à 381 patients (prévalence considérée: 55,4%) et majorée à 500 patients. Pour la seconde période (2018-2024), la taille de l'échantillon calculée sur la base de la prévalence des traitements antithrombotiques observée dans la première période était de 276 patients, et a été majorée à 500, afin d'augmenter la puissance statistique, la précision des estimations et la robustesse des analyses. Les dossiers médicaux des patients répondant aux critères d'inclusion ont été choisis selon un échantillonnage aléatoire stratifié proportionnel aux prévalences observées par année d'hospitalisation. Les strates ont été définies selon les années d'hospitalisation afin de refléter l'évolution annuelle des prescriptions et des complications liées aux antithrombotiques. Ceci a permis de garantir la représentativité temporelle de l'échantillon. L'ensemble des deux périodes a ainsi conduit à l'inclusion de 1000 patients dans notre étude.

**Variables quantitatives:** les variables quantitatives ont été exprimées sous forme de moyenne ± écart-type ou médiane (intervalle interquartile) selon leur distribution. L'âge a été analysé comme une variable quantitative, puis regroupé en deux catégories: <65 ans et ≥65 ans afin d'évaluer l'association de l'âge élevé avec la survenue de complications hémorragiques. De même, la variable nombre de traitements antithrombotiques a été groupée en deux catégories (= 1 et ≥2). Les variables qualitatives ont été exprimées sous la forme d'effectifs (n) et de pourcentages (%).

**Méthodes statistiques:** l'analyse statistique a été réalisée à l'aide du logiciel SPSS. La comparaison des variables qualitatives s'est faite selon le test du “Khi carré” et les variables quantitatives avec le test de Student, lorsque les conditions d'application étaient respectées. Le seuil de significativité statistique a été fixé à 5%. Une analyse multivariée par régression logistique a été réalisée afin d'identifier les facteurs indépendants associés à la survenue d'hémorragies chez les patients. Les variables présentant une association statistiquement significative ont été incluses dans le modèle multivarié ainsi que les variables dont le seuil de significativité était <0.2. Des variables jugées cliniquement pertinentes ont été forcées dans le modèle multivarié indépendamment de leur significativité statistique (l'HTA, la phase de l'étude: phase 1 ou phase 2). Les données manquantes, représentant moins de 5% des observations, ont été considérées comme négligeables. Les analyses ont été réalisées sans imputation.

**Aspects éthiques:** l'étude a été menée dans le respect de la confidentialité des données des patients, après approbation du comité d'éthique de l'Institut de Cardiologie d'Abidjan.

## Résultats

**Population d'étude:** au total 1380 patients hospitalisés en USIC à l'ICA entre 2013 et 2024 sur les deux périodes successives; après exclusion de 3071 dossiers (patients non cardiovasculaires, dossiers incomplets ou non disponibles), respectivement 2860 et 7870 patients étaient éligibles, parmi lesquels un échantillonnage aléatoire stratifié proportionnel a permis d'inclure 500 patients par période, soit 1000 au total. Le flux des participants à l'étude est présenté dans la Figure 1.

**Figure 1:**
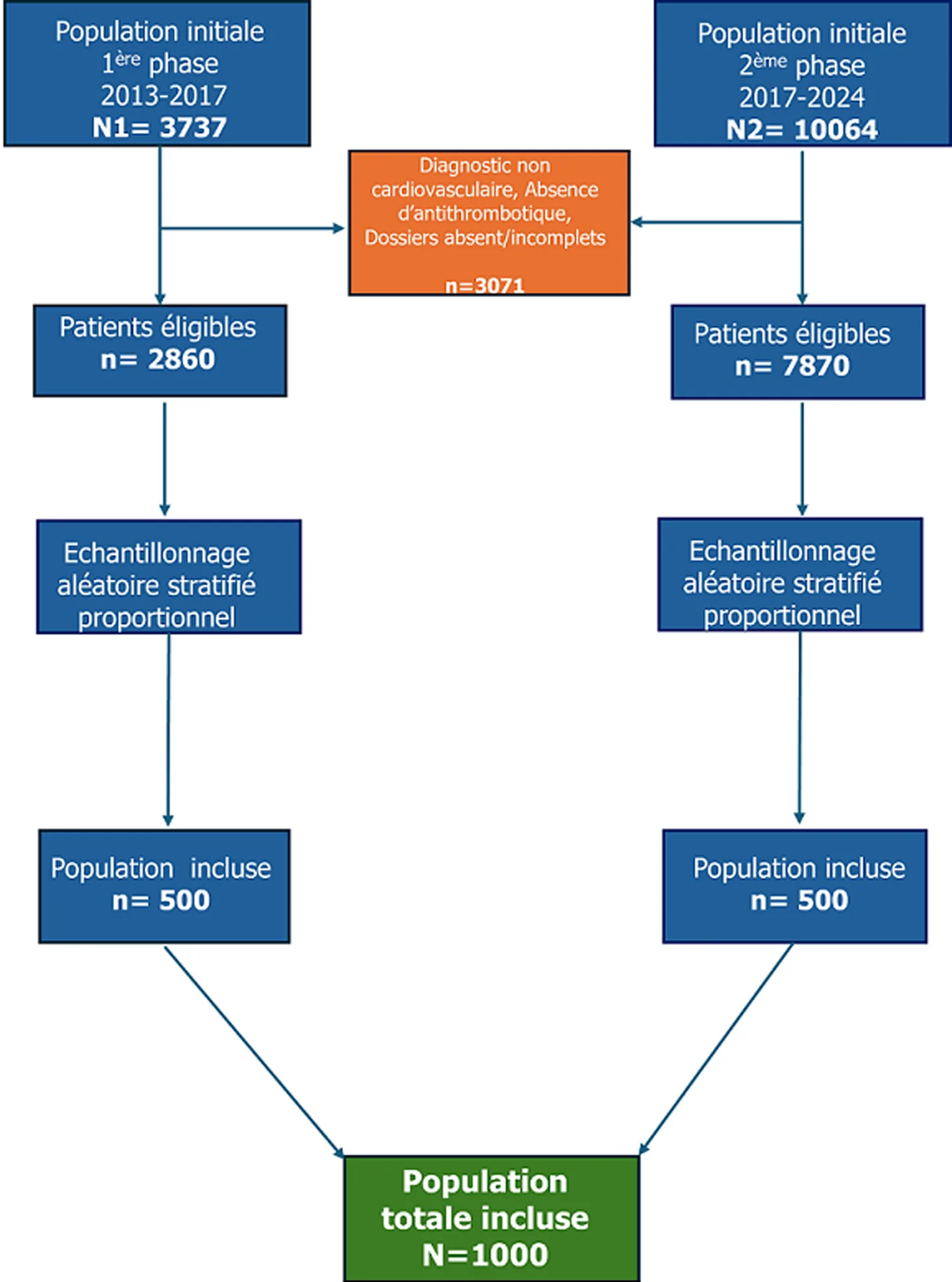
diagramme de flux de sélection de la population étudiée dans l'USIC de l'ICA

**Analyse descriptive:** on retrouvait une prédominance masculine de la population d'étude avec un sex-ratio à 1,42. L'âge moyen était de 59,5±14,1 ans. Les facteurs de risque les plus fréquemment observés dans notre population d'étude étaient l'hypertension artérielle (HTA), le tabagisme et le diabète. Le syndrome coronarien aigu constituait l'indication principale de la prescription d'un traitement antithrombotique avec une prévalence statistiquement plus élevée à la deuxième phase de l'étude (38%). Les caractéristiques de l'ensemble de nos populations sont résumées dans le Tableau 1. Sur les douze années étudiées, la proportion de patients recevant un traitement antithrombotique a augmenté de manière progressive (Figure 2), passant de 76,53% à la phase 1 à 78,65% en phase 2. Parmi les 1000 patients inclus, les données sur la survenue de complications hémorragiques étaient manquantes pour 11 patients.

**Tableau 1:** caractéristiques sociodémographiques, cliniques et thérapeutiques des patients hospitalisés en USIC à l'ICA, comparées entre la phase 1 (2013-2017) et la phase 2 (2018-2024)

Variables	Phase 1(n=500)	Phase 2(n=500)	P-value
**Age moyen(ans)**	58±15	61±13	<0,001
**Classe d'age ≥ 65ans**	151(30,2)	192(38,4)	0,007
**Sexe H/F**	290/210	297/203	0,640
**Facteurs de risque cardiovasculaire**			
HTA (%)	52% (260)	54% (270)	0,530
Tabac (%)	21% (107)	19,4% (97)	0,430
Diabète (%)	19% (97)	31% (155)	< 0,001
**Diagnostics**			
SCA (%)	29% (145)	38% (190)	0,002
Cardiopathie dilatée (%)	18% (90)	15,8% (79)	0,350
Embolie pulmonaire (%)	16,4% (82)	17,6% (88)	0,610
**Traitement antithrombotique**			
Taux prescription global (%)	76,53%	78,65%	0,040
HBPM (%)	58.5% (293)	60% (301)	0,580
HNF (%)	37,1% (186)	40,4% (202)	0,360
Aspirine (%)	34,6% (173)	33,2% (166)	0,650
Fibrinolytiques (%)	2,6% (13)	6,6% (33)	0,003
**Nombre d'antithrombotiques concomitants**			
1	25,4% (127)	18,6% (93)	0,009
≥ 2	74,6% (373)	81,4% (407)	0,009
**Complications liées au traitement antithrombotique**			
Hémorragies (%)	8% (40)	7,2% (36)	0,250
Hémorragies Mineures (%)	4,8% (24)	5,4% (27)	0,670
Hémorragies Majeures (%)	3,2% (16)	1,8% (9)	0,160
**Mortalité hospitalière (patients avec hémorragie) (%)**	37,5% (15)	30,55% (11)	0,430

USIC: unité de soins intensifs cardiologiques, ICA: l’Institut de Cardiologie d’Abidjan, H/F: hommes/femmes, HTA: hypertension artérielle, HNF: héparine non fractionnée. Les variables quantitatives sont exprimées en moyenne, écart-type et les variables catégorielles en effectifs et pourcentages. Les P-values indiquent la significativité statistique des différences entre les phases 1 et 2, au seuil alpha=5%.

**Figure 2:**
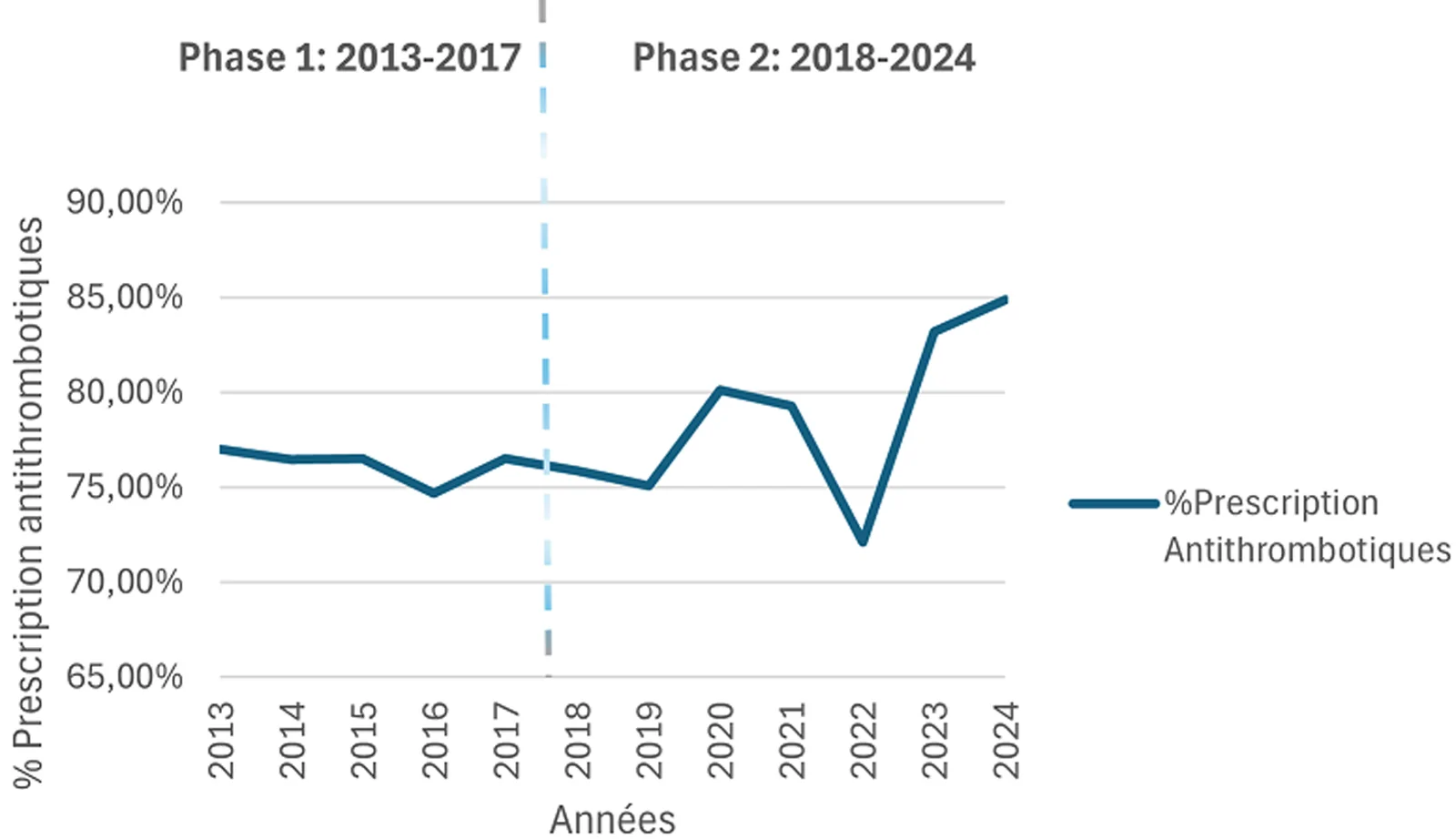
évolution annuelle des prescriptions d'antithrombotiques en USIC à l'ICA entre 2013 et 2024

**Prescription des traitements antithrombotiques et complications hospitalières:** on retrouvait ainsi une élévation statistiquement significative du taux de prescription d'antithrombotique en USIC entre la première et la seconde phase de l'étude (p=0,04). Les héparines de bas poids moléculaire demeuraient la classe d'antithrombotiques la plus prescrite au cours des deux phases (58,5% et 60%). La proportion de patients recevant une association (bi ou trithérapie antithrombotique) a augmenté de façon significative entre la première et la seconde phase de l'étude: 74,6% vs 81,4%; p=0,009. L'utilisation des fibrinolytiques a, quant à elle, fortement augmenté entre les deux phases passant de 2,6% à 6,6% avec une différence statistiquement significative entre les deux phases (Tableau 1). Les complications dues au traitement antithrombotique ont été dominées par les accidents hémorragiques. La fréquence des hémorragies était de 8% en phase 1 contre 7,2% en phase 2 de l'étude. Les hémorragies mineures (cutanées, digestives non graves) étaient les plus fréquentes. Le profil de complication est similaire entre les deux phases. L'âge a été dichotomisé en deux catégories (<65 ans et ≥65 ans), et le nombre de traitements antithrombotiques a été également catégorisé en monothérapie versus polythérapie (≥2 antithrombotiques). Il a été observé une proportion significativement plus importante d'hémorragies chez les patients âgés de ≥65 ans (82,9%). De même, les patients recevant au moins deux traitements antithrombotiques présentaient significativement plus d'événements hémorragiques (86,8%).

**Analyse multivariée:** de l'analyse multivariée par régression logistique réalisée pour identifier les facteurs indépendamment associés à la survenue d'hémorragie, il ressort que l'âge supérieur à 65 ans (OR=9,5, IC95%=5,5-16,5, p<0,001), les thrombolytiques (OR=55, IC95%=34-96, p<0,001), ainsi que l'usage concomitant d'au moins deux antithrombotiques (OR=1,6, IC95%=1,1-2,1, p=0,009), augmentent significativement le risque d'hémorragie (Tableau 2). La phase d'étude, en revanche (phase 1 vs phase 2), n'était pas un déterminant indépendant de la survenue d'hémorragie. Quant à la mortalité hospitalière, elle restait plus importante chez les patients ayant présenté un accident hémorragique mais sans différence statistiquement significative entre les deux phases de l'étude.

**Tableau 2:** facteurs indépendants associés à la survenue d'accidents hémorragiques chez les patients hospitalisés en USIC entre 2013 et 2024

Variables	Hémorragie oui (n=76)	Hémorragie non (n=924)	P-value	OR ajusté	IC 95%	P-value multivarié
**Classe d'age ≥ 65 ans**	82,9(63)	30,3(280)	<0,000	9,5	5,5- 16,5	< 0,001
**Sexe (Homme)**	48,7(37)	59,5(550)	0,007	0,9	0,61-1,2	0,65
**HTA** * **(%)**	46,05(35)	53,6(495)	0,210	0,8	0,46-1,18	0,350
**Diabète (%)**	27,-6(21)	25,0(231)	0.610			
**SCA (%)**	50,0(38)	32,1(297)	0,002	0,9	0,58-1	0,06
**Fibrinolytiques (%)**	44,7(34)	1,3(12)	<0,000	55	34-96	< 0,001
**Nombre d'antithrombotiques concomitants ≥2**	86,8(66)	77,3(714)	0,005	1,6	1,1-2,1	0,009
**Phase de l'étude** * **(phase 1 vs Phase 2)**	52,6(40)	49,8(460)	0,63	1,1	0,70-1,79	0,250

OR= Odds ratio, IC95%= Intervalle de confiance de l'odds ratio à 95%, HTA= hypertension artérielle, SCA= syndrome coronarien aigu. Les variables marquées d'un astérisque (*) ont été forcées dans le modèle de régression logistique. Les autres variables sont significatives en analyse univariée.

## Discussion

Dans notre étude, nous avons observé une population avec des caractéristiques cliniques (prévalence élevée d'HTA et de diabète) qui sont cohérentes avec les profils rapportés dans les populations à haut risque cardiovasculaire [9]. Le syndrome coronarien aigu constituait l'indication principale de prescription d'antithrombotique, cette observation étant restée stable entre les deux phases de l'étude, soulignant ainsi la constance des indications cliniques au cours des douze dernières années. L'analyse des prescriptions d'antithrombotiques a montré une tendance à l'augmentation progressive du recours aux antithrombotiques au cours de cette décennie. Cela reflète à la fois une meilleure adoption des recommandations internationales et également une prise de conscience accrue du risque thrombotique plus important chez le patient cardiovasculaire. En effet, la connaissance du risque thrombotique important chez le patient cardiovasculaire justifie la tendance à initier plus systématiquement une prophylaxie antithrombotique dans cette population [10]. Les héparines de bas poids moléculaire sont restées la classe anticoagulante la plus prescrite tandis que les associations d'antithrombotiques ont augmenté lors de la seconde phase de l'étude. On a également retrouvé une tendance marquée à l'augmentation de l'utilisation des fibrinolytiques qui peut être expliquée par une meilleure disponibilité des agents thrombolytiques, une confiance accrue des praticiens dans leur sécurité, la formation des médecins sur les indications selon les recommandations internationales et la surveillance des traitements antithrombotiques. Il reste cependant essentiel d'évaluer attentivement le rapport bénéfice/risque chez les patients âgés, comorbides qui présentent un risque hémorragique plus important [11].

En cours d'hospitalisation, 7,6% de nos patients ont présenté un accident aux antithrombotiques, à type d'accidents hémorragiques. On a observé un profil de complications resté globalement similaire entre les deux périodes. Ceci laisse suggérer que l'augmentation des prescriptions d'antithrombotiques n'a pas modifié la nature des complications observées, et ce, malgré un usage plus accru des traitements fibrinolytiques. Ce profil reste comparable à celui décrit dans la littérature où les hémorragies mineures sont les plus fréquentes [12]. Les résultats de l'analyse multivariée, à savoir les facteurs indépendants de la survenue d'accidents hémorragiques (âge avancé, fibrinolytiques et l'usage concomitant de plusieurs antithrombotiques) restent concordants avec ce qui a été rapporté par d'autres études évaluant les facteurs prédictifs d'hémorragie chez les patients sous antithrombotiques [12,13].

La mortalité hospitalière était plus élevée chez les patients ayant présenté un accident hémorragique, indépendamment de la phase d'étude, ce qui souligne l'impact pronostique des complications hémorragiques [14,15]. L'analyse montre cependant une stabilité de la mortalité dans le temps, suggérant que l'augmentation des prescriptions d'antithrombotiques, des associations bi/trithérapies antithrombotiques ne semble pas avoir impacté le pronostic global.

**Limites:** cette étude présente certaines limites, notamment le caractère rétrospectif, le caractère monocentrique et l'absence de suivi à long terme, qui peuvent restreindre la généralisation des résultats.

## Conclusion

Les traitements antithrombotiques restent très utilisés en unité de soins intensifs cardiologiques, avec une augmentation non négligeable des bithérapies et des fibrinolytiques au cours du temps. Les accidents hémorragiques, bien que surtout mineurs, continuent d'être un facteur pronostique de mortalité hospitalière. Ces constats montrent la nécessité d'une surveillance clinique et biologique rigoureuse et d'une évaluation individualisée du rapport bénéfice/risque pour améliorer la sécurité et le pronostic des patients.

### Etat des connaissances sur le sujet

- Les patients sous traitement antithrombotique sont majoritairement âgés avec des facteurs de risque cardiovasculaire;
- Les complications sous antithrombotiques les plus rapportées sont les saignements et les thrombopénies induites par l'héparine.

### Contribution de notre étude à la connaissance

- Notre étude évalue la dynamique temporelle des pratiques de prescription et des complications sous antithrombotiques en hospitalisation, en les comparant sur deux périodes successives;
- Cette étude identifie les facteurs indépendants associés aux complications hémorragiques dans un contexte d'hospitalisation pour pathologie cardiovasculaire grave.
